# Transferring Patients with Intracerebral Hemorrhage Does Not Increase In-Hospital Mortality

**DOI:** 10.1371/journal.pone.0159174

**Published:** 2016-07-28

**Authors:** Farhaan Vahidy, Claude Nguyen, Karen C. Albright, Amelia K. Boehme, Osman Mir, Kara A. Sands, Sean I. Savitz

**Affiliations:** 1 Department of Neurology, McGovern Medical School, University of Texas-Health Science Center at Houston, Houston, TX, United States of America; 2 Geriatric Research Education and Clinical Center (GRECC), Birmingham VA Medical Center, Birmingham, AL, United States of America; 3 Department of Epidemiology, School of Public Health, University of Alabama at Birmingham, Birmingham, AL, United States of America; 4 Gertrude H. Sergievsky Center, Department of Neurology, Columbia University, New York, NY, United States of America; 5 Department of Neurology, University of Alabama at Birmingham, Birmingham, AL, United States of America; University of Regensburg, GERMANY

## Abstract

**Introduction:**

Comprehensive stroke centers (CSCs) accept transferred patients from referring hospitals in a given regional area. The transfer process itself has not been studied as a potential factor that may impact outcome. We compared in-hospital mortality and severe disability or death at CSCs between transferred and directly admitted intracerebral hemorrhage (ICH) patients of matched severity.

**Materials and Methods:**

We retrospectively reviewed all primary ICH patients from a prospectively-collected stroke registry and electronic medical records, at two tertiary care sites. Patients meeting inclusion criteria were divided into two groups: patients transferred in for a higher level of care and direct presenters. We used propensity scores (PS) to match 175 transfer patients to 175 direct presenters. These patients were taken from a pool of 530 eligible patients, 291 (54.9%) of whom were transferred in for a higher level of care. Severe disability or death was defined as a modified Rankin Scale (mRS) sore of 4–6. Mortality and morbidity were compared between the 2 groups using Pearson chi-squared test and Student t test. We fit logistic regression models to estimate odds ratios (OR) and 95% confidence intervals (CI) for association between transfer status and in-hospital mortality and severe disability or death in full and PS-matched patients.

**Results:**

There were no significant differences in the PS-matched transfer and direct presentation groups. Patients transferred to a regional center were not at higher odds of in-hospital mortality (OR: 0.93, 95% CI: 0.50–1.71) and severe disability or death (OR: 0.77, 95% CI: 0.39–1.50), than direct presenters, even after adjustment for PS, age, baseline NIHSS score, and glucose on admission.

**Conclusion:**

Our observation suggests that transfer patients of similar disease burden are not at higher risk of in-hospital mortality than direct presenters.

## Introduction

Spontaneous non-traumatic intracerebral hemorrhage (ICH) accounts for 10% to 15% of all strokes; however, its mortality is greater than 30%, far exceeding that of ischemic stroke.[1–6] It has been reported that approximately 79% of ICH patients initially present to a non-tertiary care center,[7] and a significant proportion are transferred to tertiary care hospitals for higher level of care.[8] Comprehensive Stroke Centers (CSCs) are regional facilities that accept patients from non-certified hospitals, acute stroke-ready hospitals, and primary stroke centers (PSCs).[9] It is therefore possible that the most critically ill ICH patients presenting to non-CSCs are frequently transferred into CSCs because of the perceived need for advanced clinical resources, neurocritical care, neurosurgical capability, and expertise linked to better patient outcomes.[10–14]

With public reporting of hospitals focusing on in-patient mortality as an important quality metric, there is concern that a CSC’s mortality rate may be unfairly inflated against the centers that are transferring them the sickest, highest mortality-risk patients, thus “penalizing” CSCs.[15–18] One aspect that has not been fully evaluated is whether the transfer to a CSC, with its inherent time delay to intensive care, is independently associated with worse outcomes. We hypothesized that patients transferred into a CSC would have higher risk adjusted in-patient mortality and poorer functional status than patients directly presenting to a CSC. We used propensity score (PS) based methods to compare mortality and functional status between ICH patients transferred to CSC and those who presented directly.

## Materials and Methods

### Study Design and Setting

We retrospectively identified patients with spontaneous ICH at two different CSCs. Data from a prospectively collected data registry was used for site 1 [19], where as a retrospective chart review of consecutive ICH patients was performed at site 2. Both sites are Joint-Commission certified CSCs and accept transfers from surrounding hospitals; providing 24/7 vascular neurology, neurosurgery, and neurocritical care coverage with dedicated neuro-intensive care units (NICU). Requests for transfer are received by a physician who would then select the destination unit (Emergency Department [ED], NICU, stroke unit, or floor). All patients deemed to be emergent are immediately transferred.

### Study Participants

We identified all spontaneous ICH patients, age 18 and above, admitted from March 2011 to March 2012 at site 1 and between 2008 and 2013 at site 2. Patients with arteriovenous malformations (AVM), aneurysms, pure intraventricular hemorrhage (IVH), subdural hematomas (SDH), subarachnoid hemorrhage (SAH), or patients who were enrolled in an interventional clinical trial were excluded. Eligible ICH patients were categorized based on transfer status. Patients transferred into a CSC were considered exposed while patients presenting directly to a CSC were considered unexposed.

### Variables

We collected baseline demographics, including age, sex, race, past medical history, home medication use, illicit substance abuse, baseline National Institutes of Health Stroke Scale (NIHSS) score, Glasgow Coma Scale (GCS) on admission, initial imaging findings, and baseline laboratory results for patients at both participating sites.

### Measurements

#### Imaging

Initial CT scans for all patients were reviewed by two neurologists each separately at both sites, blinded to presentation group (transfers or direct presenters) and patient outcomes. Hematoma volumes were measured by the ABCD2 method.[20] Hematoma location was also designated as either supra- or infratentorial, and categorized into the following locations: cortical or lobar, subcortical (e.g., basal ganglia, thalamus), brainstem, and cerebellum. ICH score was calculated for all patients.[21]

#### Outcomes

The primary outcome of interest was all cause in-hospital mortality. This information was consistently available from either prospectively collected data registry or electronic medical records for both sites. We also collected information on discharge disposition, and functional outcome as measured by the modified Rankin Scale (mRS) score at the time of discharge or day 7 (whichever came first). Severe disability or death (SDD) was defined as a mRS score of 4–6, and has been evaluated as a secondary outcome.[22] Data on discharge disposition was available for site 1 from the stroke registry and was abstracted from medical records for site 2. Six levels of disposition were captured as discharge to home, inpatient rehabilitation, skilled nursing facility, subacute unit, transfer to another service, and hospice or death.

### Sample Size

We aimed to explore the association between in-hospital mortality and transfer to tertiary care hospitals for ICH patients. Our approach entailed assimilating all eligible ICH cases with complete data elements on all variables, over the period of investigation, from two high volume CSCs. For formal hypothesis testing, a logistic regression of a binary response variable (in-hospital mortality [Y]), on a binary independent variable (transfer [X]) with a sample size of 530 observations (of which 40% are unexposed and 60% are exposed) achieves 80% power at a 0.05 significance level to detect a change in probability (Y = 1) from the baseline value of 0.30 to 0.42. This change corresponds to an odds ratio of 1.68.[23]

### Quantitative Variables

We used propensity scores (PS) to match exposed (transfers) and unexposed (direct presenters) patients. A multivariable logistic regression model was fit to determine the PS for each patient. The PS model included baseline variables available on admission, including, but not limited to: demographics, past medical history, home medications, social history, neurologic exam, initial laboratory results, and initial imaging. A 5:1 computerized greedy matching technique, where exposed patients were first matched to unexposed patients who had a PS that was identical in all 5 digits was employed.[24] Those who did not match were then matched on 4 digits of the PS, continuing down to a 1-digit match on PS for those who remained unmatched. Balance between exposed and unexposed for the covariates in the PS-matched sample was assessed using Pearson chi-squared or independent sample Student t-test where appropriate.

### Statistical methods

Descriptive statistics were used to provide summary measures for continuous and categorical variables as means with standard deviations (SD) or medians with interquartile range (IQR) and proportions, respectively. Univariable comparisons were made between the exposed and unexposed patients; categorical variables were tested using either the Chi-Squared Test or the Fisher exact test, whereas student t-test or the Mann Whitney U test was used for continuous variables. Multivariable models were fitted to explore the association between transfer and mortality as the primary outcome, and severe disability and death (mRS 4–6) as a secondary outcome. The unadjusted odds ratios (OR) and 95% confidence interval (CI) for mortality and severe disability and death (SDD) were estimated using baseline models. These models were then sequentially risk-adjusted using PS and other important variables. Separate models were fitted for the complete data set and for a PS-matched sub-sample. Alpha was set at 0.05 for all hypothesis testing. Point estimates, confidence intervals, and p values were calculated using SAS version 9.3 (SAS Institute, Cary, NC). This study was approved by the local Institutional Review Board at each site.

## Results

### Descriptive analyses of full and matched cohorts

**Fig 1:**
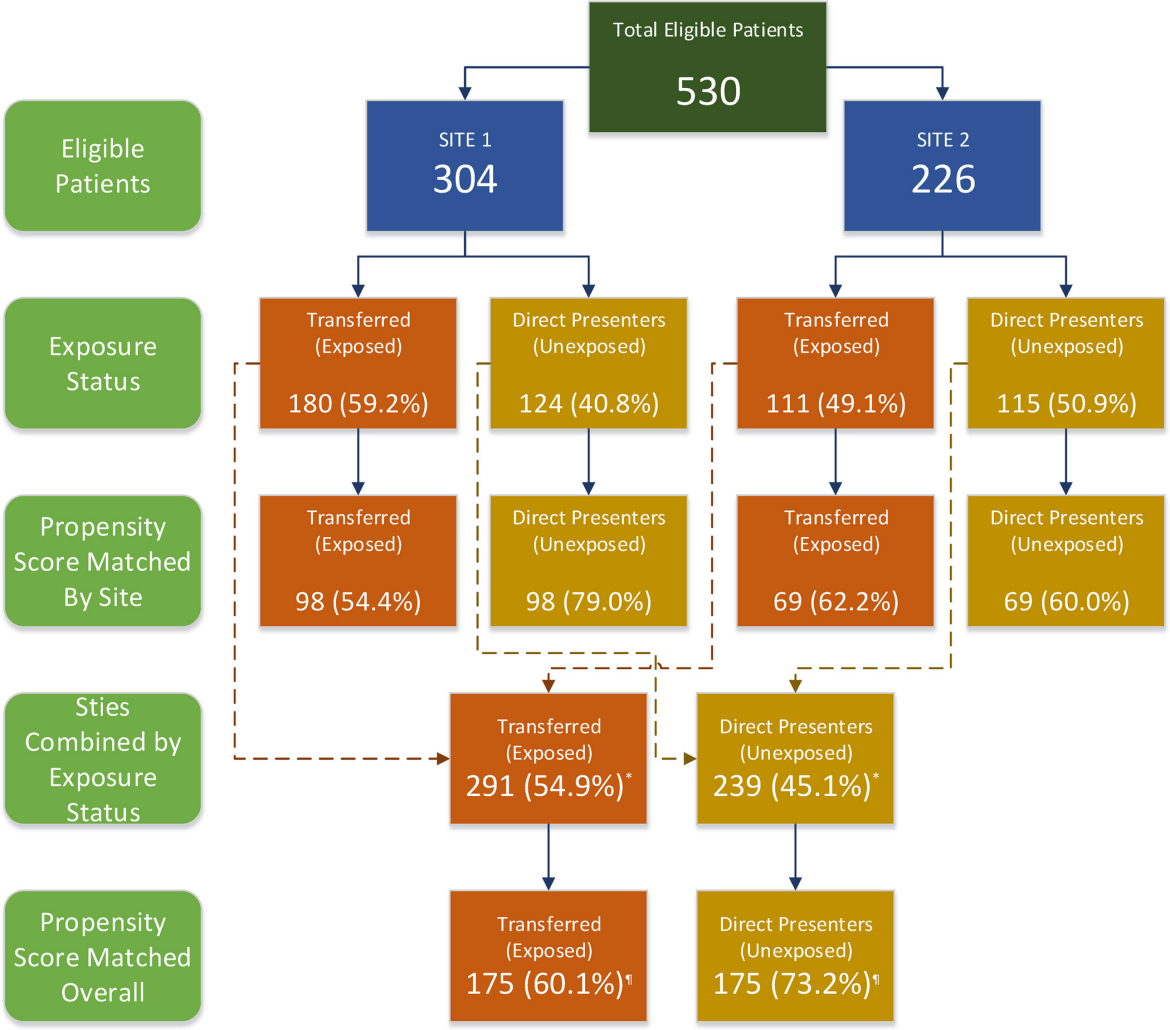
Number of transferred and directly admitted patients at participating sites before and after propensity score matching.

A total of 530 eligible ICH patients were identified from both sites. Of these, 291 (54.9%) were transferred whereas 239 (45.1%) were direct presenters. The proportion of transferred patients at site 1 was significantly higher as compared to site 2 (59.2% vs. 49.1%, p = 0.02). The number and proportion of exposed (transferred) and unexposed (direct presenters) patients at each site, before and after PS matching is presented in Fig 1.

The transferred group had significantly higher proportion of white patients. The transferred patients also had significantly lower NIHSS, higher GCS, lower ICH scores, smaller ICH volumes, and lower proportion with intraventricular extension of hemorrhage. The comparison between transferred and directly admitted patients at both sites, for important demographic, past medical history, and clinical and radiological factors at presentation is shown in Table 1.

**Table 1:** Comparison of demographic, past medical history, and clinical factors, between direct presenters and transferred patients at both sites.

Description	Presented Directly (n = 239)	Transferred In(n = 291)	p value
Age, mean (SD)	62.7 (13.6)	64.2 (14.8)	0.22
Females, n (%)	137 (57.3)	160 (54.9)	0.59
Race, n (%)			
White	119 (49.8)	192 (65.9)	<0.01
Black	104 (43.5)	78 (26.8)	
Asian	2 (0.8)	7 (2.4)	
Other	14 (5.9)	14 (4.8)	
Hispanic, n (%)	38 (15.9)	44 (15.1)	
Past Medical History, n (%)			
ICH	24 (10.0)	17 (5.8)	0.08
Ischemic stroke or TIA	22 (9.2)	45 (15.5)	0.03
Atrial Fibrillation	19 (7.9)	31 (10.7)	0.29
Diabetes Mellitus II	53 (22.2)	85 (29.2)	0.07
Hypertension	186 (77.8)	229 (78.7)	0.81
Hyperlipidemia	46 (19.3)	55 (18.9)	0.92
Coronary Artery Disease	19 (7.9)	50 (17.2)	<0.01
Peripheral Vascular Disease	3 (1.3)	7 (2.4)	0.34
Statin as a home med	41 (17.2)	48 (16.5)	0.84
History of illicit substance abuse	38 (15.9)	40 (13.8)	0.49
NIHSS on arrival, median (IQR)	16 (7–28)	9 (2–23)	<0.01
GCS on arrival, median (IQR)	11 (6–15)	14 (7–15)	0.01
Glucose on arrival, median (IQR)	133 (112–175)	130 (104–183)	0.49
Platelet count, median (IQR)	209 (164–250)	198 (163–249)	0.70
PTT, median (IQR)	29 (26–32)	29.6 (27–34)	0.055
INR, median (IQR)	1.03 (0.97–1.12)	1.08 (1.01–1.19)	<0.01
ICH Score, median (IQR)	2 (1–3)	1 (0–2)	<0.01
ICH Volume, median (IQR)	19.1 (7.7–46.1)	13 (4–35)	<0.01
ICH location, n (%)			
Cortical/Lobar	70 (29.3)	74 (25.4)	0.58
Subcortical/BG/Thalamus	145 (60.7)	184 (63.3)	
Brainstem	14 (5.7)	15 (5.2)	
Cerebellum	10 (4.2)	18 (6.2)	
IVH present, n (%)	132 (55.2)	122 (41.9)	<0.01
Comfort measures, n (%)	37 (15.5)	36 (12.4)	0.30

Using PS methods, 175 ICH patients who were transferred were matched to 175 ICH patients who presented directly from the full sample of 530 eligible patients. Baseline characteristics of the PS-matched transfer and direct presentation groups for the combined sites are shown in Table 2. PS matching balanced the important disease severity differences between exposed and unexposed patients such as presentation NIHSS, GSC, ICH Score, hematoma volume, and presence of IVH. The only difference between the PS-matched groups observed was in race with a higher proportion of the transfer patients being white (59.4% vs. 49.1%, p<0.01).

**Table 2:** Comparison of demographic, past medical history, and clinical factors, between direct presenters and transferred patients at both sites, in the propensity score–matched cohort.

Description	Presented Directly (n = 175)	Transferred In(n = 175)	p value
Age, mean (SD)	62.7 (13.5)	62.2 (15.3)	0.76
Female, n (%)	98 (56.0)	95 (54.3)	0.75
Race, n (%)			
White	86 (49.1)	104 (59.4)	<0.01
Black	80 (45.7)	52 (29.7)	
Asian	0 (0.0)	5 (2.9)	
Other	9 (5.1)	14 (8.0)	
Hispanic, n (%)	25 (14.3)	26 (14.9)	0.88
Past Medical History, n (%)			
ICH	17 (9.7)	13 (7.4)	0.45
Ischemic stroke or TIA	20 (11.4)	19 (10.9)	0.87
Atrial Fibrillation	15 (8.6)	15 (8.6)	1.00
Diabetes Mellitus II	41 (23.4)	42 (24.0)	0.90
Hypertension	47 (88.7)	45 (84.9)	0.57
Hyperlipidemia	137 (78.3)	133 (76.0)	0.61
Coronary Artery Disease	19 (10.9)	16 (9.1)	0.59
Peripheral Vascular Disease	3 (1.7)	4 (2.3)	0.70
Home medications include a statin	32 (18.3)	27 (15.4)	0.48
History of illicit substance abuse	28 (16.0)	29 (16.6)	0.89
NIHSS on arrival, median (IQR)	13 (6–25)	17 (5–27)	0.61
GCS on arrival, median (IQR)	13 (7–15)	11 (3–15)	0.27
Glucose on arrival, median (IQR)	131 (109–177)	129 (104–177)	0.52
Platelet count, median (IQR)	205 (168–250)	196 (167–248)	0.75
PTT, median(IQR)	29.0 (26. 0–31.8)	29.2 (26.0–33.9)	0.35
INR, median (IQR)	1.0 (1.0–1.1)	1.1 (1.0–1.2)	<0.01
ICH Score, median (IQR)	1 (1–3)	1 (1–3)	0.48
ICH Volume, median (IQR)	17.0 (7.7–46.1)	17.4 (6.0–41.1)	0.47
ICH Location, n (%)			
Cortical / Lobar	53 (30.3)	49 (28.0)	0.72
Subcortical / BG / Thalamus	107 (61.1)	106 (60.6)	
Brainstem	7 (4.0)	7 (4.0)	
Cerebellum	8 (4.6)	13 (7.4)	
IVH present, %	50.3 (88)	49.1 (86)	0.83
Comfort measures, %	12.0 (21)	15.4 (27)	0.35

We also performed PS matching at individual site level. These data were not found to be different from the overall matched sample (data not shown).

### Primary Outcome: In-hospital Mortality

In the overall sample of 530 patients, the proportion of in-hospital mortality was significantly higher for direct presenters as compared to those who were transferred (28.0% vs. 18.6%, p = 0.01). There was however no difference between direct and transferred patients for in-hospital mortality in the PS-matched sample (22.9% vs. 25.1%, p = 0.62). The unadjusted OR and 95% CI comparing directly admitted and transferred patients for the primary outcome of in-hospital mortality was 0.59 (0.39–0.88), suggesting a statistically significant 41% reduction in mortality for transferred patients. These estimates were no longer significantly different after controlling for PS and other important factors in both the full and the PS-matched cohorts. Table 3 illustrates the odds of in-hospital mortality for transfer patients when compared with direct presenters using multivariable models fitted for both sites combined.

**Table 3:** Odds Ratios and 95% Confidence Intervals for various multivariable models fitted to compare transferred and directly presented patients for in-hospital mortality, in the full and PS-matched cohorts.

	OR	95% CI	p value
**Full Sample (n = 530)**			
No adjustment	0.59	0.39–0.88	0.01
Adjusted for PS	0.84	0.54–1.31	0.44
Adjusted for NIHSS, age, admission glucose	0.73	0.45–1.20	0.21
Adjusted for PS, NIHSS, age, admission glucose	0.71	0.42–1.19	0.20
Adjusted for PS, site, NIHSS, age, admission glucose	0.67	0.39–1.15	0.14
**Matched Sample (n = 350)**			
No adjustment	1.13	0.69–1.85	0.62
Adjusted for PS	1.15	0.70–1.89	0.59
Adjusted for NIHSS, age, admission glucose	0.95	0.53–1.71	0.87
Adjusted for PS, NIHSS, age, admission glucose	0.94	0.52–1.69	0.83
Adjusted for PS, site, NIHSS, age, admission glucose	0.93	0.50–1.71	0.81

The unadjusted in-hospital mortality was higher for directly admitted patients as compared to transferred patients at both sites individually; however, these differences did not achieve statistical significance. (Site 1: 26.6% vs. 17.8%, p = 0.07 and Site 2: 29.6% vs. 19.8%, p = 0.09). Multivariable models were also fitted for the two sites separately, using similar adjustments as were performed for the combined data. These models did not show a statistically significant difference between transferred and directly admitted patients for in-hospital mortality (data not shown). One exception was the model for site 2 controlling for PS, NIHSS, age, and admission glucose. This model showed significantly lower odds of in-hospital mortality for transferred patients (OR, 95% CI: 0.36 (0.14–0.93).

### Secondary Outcome: Severe Disability or Death

A discharge modified Rankin Scale score of 4 to 6 was defined as severe disability or death (SDD), and was evaluated as a secondary outcome. The proportion of ICH patients with SDD was significantly higher in directly admitted patients as compared to those who were transferred (82.0% vs. 67.4%, p<0.01). This difference was also significant at both sites individually (Site 1: 90.3% vs. 79.4%, p = 0.01 and Site 2: 73.0% vs. 47.8%, p<0.01). Once matched on PS, the difference between exposed and un-exposed patients was no longer statistically significant for SDD (78.9% vs. 75.4%, p = 0.45). We also fitted multivariable models to assess the association of transfer with SDD in full and PS-matched samples, controlling for PS and other important co-variates. All models indicated lower odds of SDD associated with transfer. However, none of the point estimates were statistically significant, other than those obtained from full sample models with adjustment of PS, site, NIHSS, age, and admission glucose. The odds ratios and 95% CI obtained from these models are presented in Table 4.

**Table 4:** Odds Ratios and 95% Confidence Intervals for various multivariable models fitted to compare transferred and directly presented patients for severe disability and death, in the full and Propensity Score-matched cohorts.

	OR	95% CI	p value
**Full Sample (n = 530)**			
No adjustment	0.45	0.30–0.68	<0.01
Adjusted for PS	0.61	0.40–0.95	0.03
Adjusted for NIHSS, age, admission glucose	0.71	0.42–1.21	0.21
Adjusted for PS, NIHSS, age, admission glucose	0.58	0.33–1.02	0.06
Adjusted for PS, site, NIHSS, age, admission glucose	0.52	0.29–0.95	0.03
**Matched Sample (n = 350)**			
No adjustment	0.82	0.50–1.36	0.45
Adjusted for PS	0.82	0.50–1.37	0.45
Adjusted for NIHSS, age, admission glucose	0.79	0.43–1.47	0.46
Adjusted for PS, NIHSS, age, admission glucose	0.79	0.42–1.46	0.43
Adjusted for PS, site, NIHSS, age, admission glucose	0.77	0.39–1.50	0.44

### Other Outcomes: Discharge Disposition

A significantly higher proportion of transferred patients were discharged home as compared to directly admitted patients (26.8% vs. 13.4%, p<0.01) and likewise a higher proportion of directly admitted patients were discharged to hospice or died as compared to those who were transferred (35.2% vs. 23.0%, p<0.01). However, these differences among transferred and directly admitted patients for discharge disposition were not significant in the PS-matched sample as shown in Table 5. Table 5 also summarizes other important outcomes in the PS-matched sample.

**Table 5:** Comparison of outcomes between transferred and directly presented ICH patients at both sites after Propensity Score matching.

Outcomes	Presented Directly (n = 175)	Transferred In(n = 175)	p value
Discharge disposition, n (%)			
Home	26 (14.9)	40 (22.9)	0.12
Inpatient rehab	39 (22.3)	21 (12.0)	
Skilled Nursing Facility	13 (7.4)	14 (8.0)	
Subacute unit	3 (1.7)	4 (2.3)	
Transfer to other service	41 (23.4)	44 (25.1)	
Hospice or Death	53 (30.3)	52 (29.7)	
Discharge mRS, median (IQR)	5 (4–5)	5 (4–6)	0.87
Discharge mRS 4–6, n (%)	138 (78.9)	132 (75.4)	0.45
In-hospital mortality, n %	40 (22.9)	44 (25.1)	0.62

## Discussion

We explored association between in-hospital mortality and transfer to a higher level of care for ICH patients. Our primary outcome of interest was in-hospital mortality, as this has been a recent focus of the Centers for Medicare and Medicaid Services (CMS) in the US, for public reporting and ranking of hospitals’ quality of care. In an effort to improve in-hospital quality of care for its beneficiaries, the CMS has issued new rules that update its payment policies, under the In-patient Prospective Payment System (IPPS).[25] The new IPPS rule focuses on two primary metrics for stroke patients: 30-day unplanned readmission and 30-day death rate. The 30-day death is defined as deaths from any cause with 30 days of a hospital admission, and thus includes a measure of in-patient mortality. The lack of risk-adjustment, specifically stroke severity, in the IPPS rule for stroke mortality has been critiqued at length.[26] Although in its current form, the IPPS rule principally applies to patients with ischemic stroke, recent updates have added broader stroke related International Classification of Disease (ICD) 9 codes that can potentially capture some ICH patients as well.[27] Extension of the rule to report ICH related mortality can also be envisioned. Furthermore, in-hospital mortality continues to be an important stroke related quality of care metric used by other hospital benchmarking organizations such as University Health Systems Consortium (UHC).[28] As CSCs accept a large number of ICH patients from smaller, non-tertiary care hospitals, inflation of in-hospital mortality continues to remain a concern for physicians and hospital administrators alike.

Over time, the number of patients with ICH transferred from outside facilities has steadily increased.[8, 29] A clear understanding of the impact that the transfer process may have on patient outcome is essential given the limited advanced clinical resources, neurocritical care, neurosurgical capability, and diagnostic expertise available at the primary hospital.[30, 31] Our literature review revealed only a few small reports that have described specific transfer related parameters that may be associated with poor outcomes in ICH patients. [32, 33] We hypothesized that ICH patients transferred to CSCs could have higher in-hospital mortality than direct presenters. Our hypothesis was based upon assumption that the inherent delay in receiving intensive care could be associated with increased mortality risk. For example, it is conceivable that blood pressure control is highly variable and is not as tightly regulated during the transfer process when compared to patients directly admitted to CSCs. To address this hypothesis, we used propensity score matching to compare direct presenters and transfer patients with similar demographics, comorbidities, and ICH severity. Using this methodology, we observed no difference in mortality between those who were transferred for a higher level of care and those who presented directly to our two tertiary care centers. Therefore, we found no evidence that patients transferred for a higher level of care had higher odds of mortality when compared to matched direct presenters.

A prior study from a single tertiary care hospital, of 77 direct presenters and 48 transferred ICH patients, reported a significantly greater proportion of directly admitted patients with better outcome (mRS 0–3) at hospital discharge.[34] However, the investigators did not find any differences for in-hospital mortality between the two groups (22.1% vs. 22.9%, p = 0.91). Although our results corroborate with this report in terms of in-hospital mortality, our data did not show worse outcomes (mRS 4–6) associated with transfer. Differences in patient population, pre-tertiary hospital care, and regional referral patterns may account for this variation. Our findings are also similar to another report from larger national inpatient sample database, that showed no difference between transferred and non-transferred ICH patients for in-hospital mortality.[35] However, lack of stroke specific disease severity measures may not readily permit adequate adjustments in such administrative databases.[36]

Unlike previous studies, the unique nature of the PS method allowed us to match direct presenters and transfers on the probability of being transferred, controlling for factors known at the time that the decision was made to transfer to a tertiary care center.

Our findings should be interpreted in the context of its known and potential limitations. Our small sample size may have limited our ability to detect differences between groups; however, a sample size of 175 matches is seen as reasonable for PS methodology.[37, 38] This study focuses on the short-term outcomes of ICH patients cared for at two tertiary care facilities. We do not have information on all ICH patients presenting to acute care hospitals in each of our catchment areas. We report our observations as tertiary care centers receiving patients in transfer, not as centers transferring patients out for a higher level of care. Unfortunately, the lack of granularity in outside medical records prevents us from reporting on management parameters at referring hospitals, the amount of time spent at outside facilities, the amount of time spent in transit from the transferring hospital to the receiving hospital, and the relationship between these blocks of time and short-term mortality. Finally, our findings may not be readily generalizable to other CSCs or hospital systems, based on regional differences in patient populations and referral patterns.

Despite these limitations, we feel that this analysis contributes to the literature in two ways: (1) it demonstrates that the process of transfer, in-and-of itself, does not appear to increase in-hospital mortality in ICH patients, (2) it also serves the important role of starting a dialogue about the implications of using short-term mortality without adjustment for ICH severity as a quality metric in ICH patients. Further prospective research is needed to determine if transfer is truly associated with higher risk of in-hospital mortality in ICH patients. Variations in provision of initial care and independence of hospital systems are some of the challenges in this regard. We propose harnessing the potential of national and regional stroke research consortia to assimilate cohorts of ICH patients, at various levels of care, that allow for collection of patient level outcomes data, including transfer metrics. If our results are replicated in larger samples and different sites, it would suggest that the transfer process does not pose increased harm to patients with ICH who are being referred to a more advanced center for a higher level of care.

## References

[1] Go AS, Mozaffarian D, Roger VL, Benjamin EJ, Berry JD, Blaha MJ, et al Heart Disease and Stroke Statistics—2014 Update: A Report From the American Heart Association. Circulation. 2013 10.1161/01.cir.0000441139.02102.80 . 24352519 PMC5408159

[2] Broderick J, Brott T, Tomsick T, Tew J, Duldner J, Huster G. Management of intracerebral hemorrhage in a large metropolitan population. Neurosurgery. 1994;34(5):882–7; discussion 7. . 8052387 10.1227/00006123-199405000-00015

[3] Dennis MS. Outcome after brain haemorrhage. Cerebrovascular diseases. 2003;16 Suppl 1:9–13. doi: 69935. . 12698013 10.1159/000069935

[4] Flaherty ML, Haverbusch M, Sekar P, Kissela B, Kleindorfer D, Moomaw CJ, et al Long-term mortality after intracerebral hemorrhage. Neurology. 2006;66(8):1182–6. 10.1212/01.wnl.0000208400.08722.7c . 16636234

[5] Fogelholm R, Murros K, Rissanen A, Avikainen S. Long term survival after primary intracerebral haemorrhage: a retrospective population based study. Journal of neurology, neurosurgery, and psychiatry. 2005;76(11):1534–8. 10.1136/jnnp.2004.055145 16227546 PMC1739413

[6] Special report from the National Institute of Neurological Disorders and Stroke. Classification of cerebrovascular diseases III. Stroke. 1990;21(4):637–76. 10.1161/01.str.21.4.637 2326846

[7] Adeoye O, Haverbusch M, Woo D, Sekar P, Moomaw CJ, Kleindorfer D, et al Is ED disposition associated with intracerebral hemorrhage mortality? The American journal of emergency medicine. 2011;29(4):391–5. Epub 2010/09/10. 10.1016/j.ajem.2009.10.016 20825807 PMC3005610

[8] Vahidy F, Albright K, Donnelly JP, Shapshak AH, Savitz SI. Abstract WMP31: National Trends in Transfer of Patients With Intracerebral Hemorrhage to Teaching Hospitals. Stroke. 2016;47(Suppl 1):AWMP31–AWMP.

[9] Alberts MJ, Latchaw RE, Selman WR, Shephard T, Hadley MN, Brass LM, et al Recommendations for comprehensive stroke centers: a consensus statement from the Brain Attack Coalition. Stroke. 2005;36(7):1597–616. 10.1161/01.STR.0000170622.07210.b4 . 15961715

[10] Bardach NS, Zhao S, Gress DR, Lawton MT, Johnston SC, Fisher WS. Association Between Subarachnoid Hemorrhage Outcomes and Number of Cases Treated at California Hospitals * Editorial Comment. Stroke; a journal of cerebral circulation. 2002;33(7):1851–6. 10.1161/01.str.0000019126.43079.7b 12105365

[11] Glance LG, Li Y, Osler TM, Dick A, Mukamel DB. Impact of patient volume on the mortality rate of adult intensive care unit patients. Critical care medicine. 2006;34(7):1925–34. 10.1097/01.CCM.0000226415.93237.84 . 16715030

[12] Diringer M, Edwards D. Admission to a neurologic/neurosurgical intensive care unit is associated with reduced mortality rate after intracerebral hemorrhage. Critical care medicine. 2001;29(3):635–40. PubMed Central PMCID: PMC11373434. 11373434 10.1097/00003246-200103000-00031

[13] Mirski MA, Chang CW, Cowan R. Impact of a neuroscience intensive care unit on neurosurgical patient outcomes and cost of care: evidence-based support for an intensivist-directed specialty ICU model of care. Journal of neurosurgical anesthesiology. 2001;13(2):83–92. . 11294463 10.1097/00008506-200104000-00004

[14] Suarez JI, Zaidat OO, Suri MF, Feen ES, Lynch G, Hickman J, et al Length of stay and mortality in neurocritically ill patients: impact of a specialized neurocritical care team. Critical care medicine. 2004;32(11):2311–7. 10.1097/01.ccm.0000146132.29042.4c . 15640647

[15] Rockson SG, Albers GW. Comparing the guidelines: anticoagulation therapy to optimize stroke prevention in patients with atrial fibrillation. Journal of the American College of Cardiology. 2004;43(6):929–35. 10.1016/j.jacc.2003.11.028 . 15028346

[16] Reeves MJ, Wehner S, Organek N, Birbeck GL, Jacobs BS, Kothari R, et al Accuracy of identifying acute stroke admissions in a Michigan Stroke Registry. Preventing chronic disease. 2011;8(3):A62 Epub 2011/04/12. 21477502 PMC3103567

[17] Sacco RL, Adams R, Albers G, Alberts MJ, Benavente O, Furie K, et al Guidelines for prevention of stroke in patients with ischemic stroke or transient ischemic attack: a statement for healthcare professionals from the American Heart Association/American Stroke Association Council on Stroke: co-sponsored by the Council on Cardiovascular Radiology and Intervention: the American Academy of Neurology affirms the value of this guideline. Stroke. 2006;37(2):577–617. 10.1161/01.STR.0000199147.30016.74 . 16432246

[18] Centers for M, Medicaid Services HHS. Medicare Program; hospital inpatient prospective payment systems for acute care hospitals and the long-term care hospital prospective payment system changes and FY2011 rates; provider agreements and supplier approvals; and hospital conditions of participation for rehabilitation and respiratory care services; Medicaid program: accreditation for providers of inpatient psychiatric services. Final rules and interim final rule with comment period. Federal register. 2010;75(157):50041–681. . 20712087

[19] Rahbar MH, Gonzales NR, Ardjomand-Hessabi M, Tahanan A, Sline MR, Peng H, et al The University of Texas Houston Stroke Registry (UTHSR): implementation of enhanced data quality assurance procedures improves data quality. BMC neurology. 2013;13(1):61 10.1186/1471-2377-13-61 23767957 PMC3687564

[20] RU K, Brott TG. The ABCs of measuring intracerebral hemorrhage volumes. Stroke. 1996;27(8):1304–5. 8711791 10.1161/01.str.27.8.1304

[21] Hemphill JC, Bonovich DC, Besmertis L, Manley GT, Johnston SC, Tuhrim S. The ICH Score: A Simple, Reliable Grading Scale for Intracerebral Hemorrhage Editorial Comment: A Simple, Reliable Grading Scale for Intracerebral Hemorrhage. Stroke. 2001;32(4):891–7. 10.1161/01.str.32.4.891 11283388

[22] Savitz SI, Lew R, Bluhmki E, Hacke W, Fisher M. Shift Analysis Versus Dichotomization of the Modified Rankin Scale Outcome Scores in the NINDS and ECASS-II Trials. Stroke. 2007;38(12):3205–12. 10.1161/strokeaha.107.489351 17975102

[23] Hsieh FY, Bloch DA, Larsen MD. A simple method of sample size calculation for linear and logistic regression. Statistics in medicine. 1998;17(14):1623–34. 9699234 10.1002/(sici)1097-0258(19980730)17:14<1623::aid-sim871>3.0.co;2-s

[24] Parsons L. Reducing Bias in a Propensity Score Matched-Pair Sample Using Greedy Matching Techniques. SAS Users Group International. 2004;26:214–26.

[25] States FGotU. Medicare Program; Hospital Inpatient Prospective Payment Systems for Acute Care Hospitals and the Long-Term Care Hospital Prospective Payment System and Fiscal Year 2014 Rates; Quality Reporting Requirements for Specific Providers; Hospital Conditions of Participation; Payment Policies Related to Patient Status 2016 [cited 2016 June 14]. 78 FR 50495]. Available from: https://www.federalregister.gov/articles/2013/08/19/2013-18956/medicare-program-hospital-inpatient-prospective-payment-systems-for-acute-care-hospitals-and-the. 23977713

[26] Fonarow GC, Alberts MJ, Broderick JP, Jauch EC, Kleindorfer DO, Saver JL, et al Stroke Outcomes Measures Must Be Appropriately Risk Adjusted to Ensure Quality Care of Patients A Presidential Advisory From the American Heart Association/American Stroke Association. Stroke. 2014;45(5):1589–601. 10.1161/STR.0000000000000014 24523036

[27] Services CfM, Medicaid. Measure Methodology 2016 [updated 06/09/2016 6:23 AM]. Available from: https://www.cms.gov/Medicare/Quality-Initiatives-Patient-Assessment-Instruments/HospitalQualityInits/Measure-Methodology.html.

[28] UHC for Me™ 2016. Available from: https://www.uhc.edu/Home.

[29] Albright KC, Boehme AK, Mullen MT, Seals S, Grotta JC, Savitz SI. Changing demographics at a comprehensive stroke center amidst the rise in primary stroke centers. Stroke; a journal of cerebral circulation. 2013;44(4):1117–23. 10.1161/STROKEAHA.111.666156 . 23412378 PMC4778254

[30] Albright KC, Branas CC, Meyer BC, Matherne-Meyer DE, Zivin JA, Lyden PD, et al ACCESS: acute cerebrovascular care in emergency stroke systems. Archives of neurology. 2010;67(10):1210–8. Epub 2010/10/13. 10.1001/archneurol.2010.250 . 20937948

[31] Ward MJ, Shutter LA, Branas CC, Adeoye O, Albright KC, Carr BG. Geographic access to US Neurocritical Care Units registered with the Neurocritical Care Society. Neurocritical care. 2012;16(2):232–40. 10.1007/s12028-011-9644-2 . 22045246 PMC5769870

[32] Kodankandath T, Shaji J, Pishanidar S, Kohn N, Sotudeh N, Wright P. Inconsistent Systolic Blood Pressure Management and Poor Documentation During Transfer to a Comprehensive Stroke Center Is Associated with Worsening Patient Outcomes (P5. 216). Neurology. 2016;86(16 Supplement):P5. 216.

[33] Tadevosyan A, Chen P, Anandaram S, Rojas-Soto D, Lukovits T. Abstract TP333: Independent Variables Contributing to Stroke Mortality in a Tertiary Medical Center With a Large Rural Referral Base. Stroke. 2016;47(Suppl 1):ATP333–ATP.

[34] Naval NS, Carhuapoma JR. Impact of pattern of admission on ICH outcomes. Neurocritical care. 2010;12(2):149–54. 10.1007/s12028-009-9302-0 . 19915983

[35] Adil MM, Prabhakaran S. Abstract T P243: Mortality Among Transferred Hemorrhagic Stroke Patients in United States: An Analysis From National Database. Stroke. 2014;45(Suppl 1):ATP243–ATP.

[36] Aylin P, Bottle A, Majeed A. Use of administrative data or clinical databases as predictors of risk of death in hospital: comparison of models. Bmj. 2007;334(7602):1044 Epub 2007/04/25. 10.1136/bmj.39168.496366.55 17452389 PMC1871739

[37] Quigley DD. Using multivariate matched sampling that incorporates the propensity score to establish a comparison group: Center for the Study of Evaluation, National Center for Research on Evaluation, Standards, and Student Testing, Graduate School of Education & Information Studies, University of California, Los Angeles; 2003.

[38] Holmes W, Olsen L, editors. Using propensity scores with small samples. annual meetings of the American Evaluation Association San Antonio, Texas; 2010.

